# Precision psychiatry: thinking beyond simple prediction models – enhancing causal predictions: commentary, Seyedsalehi et al

**DOI:** 10.1192/bjp.2025.10473

**Published:** 2025-11-13

**Authors:** Aida Seyedsalehi, Giulio Scola, Seena Fazel

**Affiliations:** 1 Department of Psychiatry, University of Oxford, Oxford, UK; 2 Oxford Health NHS Foundation Trust, Oxford, UK

The Feature article by Krishnadas and colleagues^1^ brings a welcome focus on causal prediction modelling — an emerging field at the intersection of prediction research and causal inference that enables risk prediction under hypothetical interventions — and provides several helpful insights into the potential for these models to aid clinical decision-making and enable more targeted intervention. However, in our view, the discussion around some of the issues covered in the original article requires more nuance, and the conclusion that any non-causal prediction model ‘may be futile at best and actively harmful at worse’ is overstated and misleading.

First, Krishnadas et al. have highlighted the inability to establish causation as a limitation of current prediction models in psychiatry. However, the aim of clinical prediction models is not to establish whether intervening on a risk factor would change the outcome value at an individual level; it is to predict an individual’s probability of the outcome given a set of covariates, which may or may not be causally related to the outcome. Therefore, the underlying causal structure of the data — identifying confounders, colliders, mediators, etc. — is not the focus of prediction modelling research. It is also important to highlight that beyond informing decision-making about treatment and resource allocation — one major role for prediction models and the focus of Krishnadas et al.’s piece — these models have other important applications in clinical practice and medical research, including providing information on prognosis to patients and clinicians and assisting with patient selection and statistical analysis in randomised controlled trials.^2^

Second, the suggestion that prediction models cannot be actionable without capturing the causal relationships between predictors and the outcome is not entirely accurate. While we agree that a predicted probability alone does not guide the choice of which specific treatment to recommend to an individual, it can identify whether this person is at high risk of a poor outcome, and would therefore benefit from additional preventive or therapeutic interventions.^2^ Recommending an intervention known to reduce the outcome risk at the population level (based on the estimated average treatment effect) to individuals identified at high absolute risk of the outcome (based on the model’s predictions) is valuable from a personalised medicine perspective. At the same time, we acknowledge that there are clinical applications for which the prediction question of interest involves estimating risk under hypothetical interventions (i.e. ‘what-if’ questions). Where this is the case, methods from causal inference are required to enable counterfactual predictions, and the Feature article has provided a useful overview of these approaches.

A broader issue, which applies to all clinical prediction models, whether causal or not, is that the prediction question of interest should be clearly defined in terms of how it relates to treatment. For example, is the clinician using the model interested in the patient’s risk assuming no treatment is given (i.e. untreated risk), their risk under current standard care, or something else?^3^ Most prediction models are developed using datasets in which patients can receive various treatments (that modify outcome risk) during follow-up. As has been recently proposed in the ‘predictimand’ framework by van Geloven et al.,^3^ the way post-baseline treatment is handled during model development will affect the interpretation of the resulting predictions and how the model can be applied in practice. For instance, a prediction model developed using standard (non-counterfactual) methods, which ignores treatment initiation after baseline, is predicting risk under the treatment assignment policy inherent to the development data (and will only be generalisable to new patients if the application setting has similar treatment practices).^3^ Such predictions can be useful if the aim is to identify patients at elevated risk of experiencing the outcome under current standard treatment, who would benefit from additional interventions. As an example, the Oxford Mental Illness and Violence (OxMIV) tool, which estimates 1-year risk of violent offending in individuals with severe mental illness and has been validated in UK Early Intervention for Psychosis (EIP) services,^4^ can support clinicians to identify high-risk individuals for further assessment or offering additional interventions to target modifiable risk factors. These could include treating co-occurring substance misuse, allocating additional resources to achieve better control of psychotic symptoms (e.g. increased frequency of follow-up visits and medication reviews), considering additional psychological therapy to improve insight and therapeutic disengagement, or addressing environmental risk factors such as unstable housing.^4^ In self-harm, structured approaches and risk prediction models can underscore safety planning, the need for further psychosocial assessment, and improve risk communication within and between services.^5^ However, in settings where the aim is to predict risk under various hypothetical treatment scenarios (e.g. no treatment, treatment A, or treatment B) in order to make a choice between them, the question of interest involves counterfactual prediction; we agree that these questions can only be answered using causal methods.

While causal prediction modelling offers opportunities for precision psychiatry, there are also important challenges. As noted in the piece, valid estimation of counterfactual predictions from observational data requires expert knowledge and strong and untestable assumptions.^6^ In particular, the exchangeability assumption requires that all covariates that independently affect both treatment assignment and the outcome have been measured and appropriately adjusted for.^6^ While expert knowledge is essential to enhance the plausibility of the exchangeability assumption (conditional on the measured covariates), there is no guarantee that this assumption holds, as there may be unknown confounders, and data on some known confounders may not be available in the model development dataset.^6^ For time-varying treatments, satisfying the exchangeability assumption is even more challenging, as one may need to adjust for time-varying confounders (i.e. time-dependent variables that affect both subsequent treatment and the outcome, and may themselves be affected by past treatment).^7^ Correctly estimating causal effects in such settings requires repeated measurements of both treatment status and all relevant time-dependent confounders, which may not be available in the development dataset. Furthermore, the statistical methods that are needed to appropriately account for time-varying confounders affected by past treatment (i.e. g-methods)^6^ can be challenging to apply in practice and their implementation requires statistical expertise.^7^

Finally, the discussion of causal predictions by Krishnadas et al. is confined to model development. Evaluating the predictive performance of counterfactual prediction models (i.e. validation) is a key challenge, since it is impossible to observe the full set of potential outcomes for all patients in any observational validation dataset.^3^ Validating predictions under hypothetical interventions has received much less attention in the field than model development, although methods have recently been proposed for both binary and time-to-event outcomes.^8^

Causal prediction models represent an important path for future prediction modelling research in mental health, with potential applications to augment clinical prognosis and in precision psychiatry more generally. However, the most appropriate method for developing prediction models will depend on the application setting and the prediction question of interest. Regardless of the approach used, it is essential that researchers explicitly specify the prediction estimand of their model (i.e. the quantity that the model is targeting) and that this reflects the intended use of the model for future patients.^3^ It is also important that the interpretation of the resulting predictions is communicated clearly to potential model users, and that the coefficients from a factual prediction model are not interpreted causally^9^ (e.g. it is not possible to obtain estimates of hypothetical risk by inputting values for the hypothetical treatment via baseline covariates). In our view, increasing awareness of the principles and methods of counterfactual prediction modelling among researchers will also lead to an improved understanding of what factual prediction models can and cannot do, minimising the potential for harm as a result of misinterpretation and misuse of these models. Prediction modelling holds considerable promise in mental health, in relation to diagnosis, prognosis, and treatment allocation, and high-quality methods should be prioritised in future research.

## Data Availability

Data availability is not applicable to this article as no new data were created or analysed in this study.
